# Multi-Faceted Constructs in Abnormal Psychology: Implications of the Bifactor S - 1 Model for Individual Clinical Assessment

**DOI:** 10.1007/s10802-020-00624-9

**Published:** 2020-02-21

**Authors:** Michael Eid

**Affiliations:** Department of Psychology, Free University of Berlin(Freie Universität Berlin), Habelschwerdter Allee 45, 14195 Berlin, Germany

**Keywords:** Bifactor model, Bifactor S-1 model, Clinical assessment

## Abstract

Burns et al. (this issue) have shown that the application of the symmetrical bifactor model to attention-deficit/hyperactivity disorder (ADHD) and oppositional defiant disorder (ODD) symptoms leads to anomalous and inconsistent results across different rater groups. In contrast to the symmetrical bifactor model, applications of the bifactor S-1 model showed consistent and theoretically well-founded results. The implications of the bifactor S-1 model for individual clinical assessment are discussed. It is shown that individual factor scores of the bifactor S-1 model reveal important information about the profile of individual symptoms that is not captured by factor scores of the multidimensional model with correlated first-order factors. It is argued that for individual clinical assessment factor scores from both types of model (multidimensional model with correlated first-order factors, bifactor S -1 model) should be estimated and compared. Finally, a general strategy for choosing an appropriate model for analyzing multi-faceted constructs is presented that compares areas of applications for (1) the multidimensional model with correlated first-order factors, (2) the bifactor S-1 model with a general reference factor, and (3) the bifactor S – 1 model with a directly assessed general factor.

Like in many other areas of psychological assessment the bifactor model has gained increasing interest in clinical psychology in recent years (Markon 2019). Although the model is more than 80 years old (Holzinger and Swineford 1937) it has been leading a niche existence for a long time and was rediscoverd only about ten years ago (Eid et al. 2017; Reise 2012). Over the last decade, however, it has become very popular and is widely used for analyzing multidimensional data. Its application is mainly based on the idea that dimensions that are correlated have something in common and this common part should be represented by a general factor. According to the basic idea of the bifactor model, an observed variable (e.g., clinical scale) can be decomposed into three parts: (1) A part shared with all other scales (represented by the general factor), (2) and a part that is only shared with the other scales representing the same facet of a construct, but not the scales representing other facets (represented by a facet-specific factor), and (3) measurement error. Based on this decomposition, several interesting research questions can be analyzed, for example, whether the general factor is sufficient to predict other phenomena or to which degree specific factors contribute to predicting phenomena beyond the general factor (Eid et al. 2018).

In many applications, the bifactor model is not based on a strong theoretical definition of the general factor but it is applied in a more exploratory way to find out empirically what a general factor might mean. Unfortunately, different empirical applications of the bifactor model in the same area of research have not resulted in clear results. A prominent example in abnormal psychology is the general factor of psychopathology (p factor; Caspi and Moffitt 2018; Lahey et al. 2012). After reviewing the research on the p factor of psychopathology over the last eight years, Watts et al. (2019) concluded that “the precise nature of the p factor is not yet understood” (p. 1285). It is only recently that systematic analyses of applications of the bifactor model revealed that many applications are affected by serious problems (e.g., negative variances of specific factors, vanishing specific factors, irregular loadings patterns; see, for example, Eid et al. 2017). Moreover, the results of these applications are typically not in line with the theoretical expectations. For example, Watts et al. (2019) convincingly stated that from a theoretical point of view all observed variables of a bifactor model should have relatively equal loadings on the common general factor and each facet should be represented by a specific factor. This structure, however, is often not supported (Eid et al. 2017; Watts et al. 2019). The many estimation problems and the theoretically not expected results found in many applications show that the bifactor model is not a reasonable model for analyzing multi-faceted constructs in clinical psychology (Heinrich et al. 2018; Markon 2019; Watts et al. 2019). A major reason for the unsatisfactory results in clinical psychology is that the facets of clinical symptoms typically are not interchangeable as it is required for a psychometrically valid application of a bifactor model (Eid et al. 2017).

Burns, Geiser, Servera, Becker, and Beauchaine (this issue) discuss and illustrate the problems that are related to the application of the bifactor model in abnormal psychology referring to attention-deficit/hyperactivity disorder (ADHD) and oppositional defiant disorder (ODD) symptoms. They give a thorough overview of 22 recent applications of the bifactor model to analyzing the structure of ADHD/ODD symptoms. They show that also in this important area of abnormal child psychology the typical application problems of the bifactor model show up. From a theoretical point of view it is particularly problematic that the results are very inconsistent across the different applications and do not allow to give the general factor a clear meaning. The statement of Watts et al. (2019) that “the precise nature of the p factor is not yet understood” (p. 1285) refers in an analogous way to the general factor of ADHD/ODD symptoms.

In contrast to the many problematic applications of the bifactor model, Burns et al. (this issue) convincingly show that the application of the bifactor S -1 model (Eid et al. 2017) is not affected by these problems and leads to consistent results across different assessment methods of ADHD/ODD symptoms. In the bifactor S -1 model, the general factor is defined by the indicators of one facet that is taken as reference facet. The indicators of the reference facets have loadings only on the general factor whereas the items of all other facets load on the general factor as well as a group factor that is specific to all indicators of a facet (called specific residual factor by Burns et al.) (see Fig. 2b). The specific residual factors, can be correlated. It is a major strength of Burn et al.’s applications of the bifactor S-1 model that the reference facet is chosen based on strong theoretical arguments. This gives the general factor as well as the specific factor a clear meaning. Moreover, the meanings of the general (reference) factor and the specific factors did not differ between the three different rater groups analyzed by Burns et al., which was not the case when applying the original bifactor model (which is called symmetrical bifactor model by Burns et al.). Choosing this reference factor in all future applications of the bifactor S-1 model would ensure that the meaning of the general factor does not change between studies, which was not the case in the 22 previous application of the bifactor model to ADHD/ODD symptoms. This makes it possible to use the bifactor S-1 model – in contrast to the traditional (symmetrical model) – for individual clinical assessment. To the best of my knowledge the implications of the bifactor S-1 model for individual clinical assessment has not be discussed so far. In my comment on the article of Burns et al. (this issue), I will focus on the implications of the bifactor S-1 model for individual clinical assessment. I will put this in a broader context of linking psychometric modeling of multi-faceted constructs and individual clinical assessment, and illustrate the implications referring to the ADHD/ODD symptoms considered by Burns et al.. Finally, I will propose a strategy for how multidimensional models can generally be used for the assessment of multi-faceted constructs.

## Psychometric Modeling of Multi-Faceted Constructs: Implications for Individual Clinical Assessment

Psychometric models – like the bifactor model – have at least two functions. First, they allow researchers to test theoretical assumptions about the structure of observed variables and to estimate parameters that are important for evaluating the quality of assessment instruments (e.g., reliability). Second, they are measurement models that allow estimating individual scores on the latent variables that can be used for individual assessment. For example, models of item response theory are not only applied to analyze the structure of test items but also to estimate, for example, ability scores and the precision with which this scores can be estimated (e.g., Van der Linden and Hambleton 1997). It is a sign of high quality when individual assessment is based on estimated scores of latent variables of a well-fitting psychometric model.

The many applications of the bifactor model pursued the first purpose of psychometric models and aimed at analyzing the structure of a multi-faceted construct. Given the many inconsistent results of applications of the bifactor model it would not be reasonable to use this model for clinical assessment. In order to use a psychometric model for individual clinical assessments it is necessary to show that applications of the model to different samples result in consistent findings.

It is a strong merit of the study of Burns et al. (this issue) that they show that applications of the bifactor S-1 model to different rater groups led to consistent results. If future applications of the bifactor S-1 model to ADHD/ODD symptoms show consistent results this will be an important cornerstone for using this model for individual clinical assessment. The bifactor S-1 model can complement clinical assessment in an important way as it provides a clinician with important information that is not provided by other psychometric models.

**Fig. 1:**
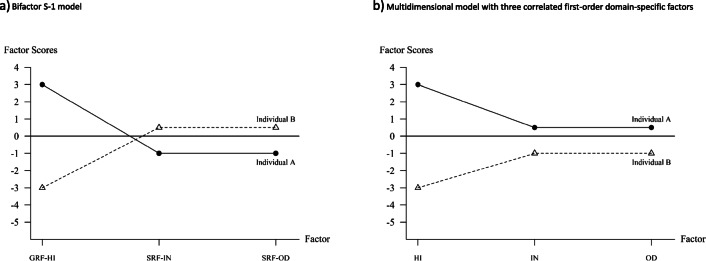
Individual factor scores for two individuals according to (**a**) the bifactor S-1 model presented by Burns et al. (this issue) with hyperactivity-impulsivity (HI) as general reference factor (GRF-HI) and two specific reference factors for inattention (SRF-IN) and oppositional defiant disorder (SRF-OD) (see Fig. 2b), and (**b**) a multidimensional model with correlated first-order facet-specific factors (see Fig. 2a) for hyperactivity-impulsivity (HI), inattention (IN), and oppositional defiant disorder (OD). All factors are centered (mean of 0). The factor scores of the model in the multidimensional model are calculated based on the regression eqs. IN = 0.6 ·GRF-HI + SRF-IN and OD = 0.6 ·GRF-HI + SRF-OD. The regression coefficient of 0.6 was roughly based on the results in Burns et al. (this issue)

In Fig. 1a the factor scores of two individuals with respect to the bifactor S-1 model presented by Burns et al. (Fig. 1b in their paper) are presented. The figure is based on centered factors (factor means of 0) which is the default setting of many programs for confirmatory factor analysis (CFA). Individual A (black dots) has a value of 3 on the general reference factor, that means that the hyperactivity-impulsivity score is above average. The scores on the specific factors are both −1 for individual A. This means that the inattention and oppositional defiant scores of Individual A are lower than expected given the hyperactivity-impulsivity score of this individual. Compared to all other individuals with the same hyperactivity-impulsivity score the inattention and oppositional defiant scores are below average, Individual A has comparably less problems with respect to inattention and oppositional defiant disorder symptoms. Individual B, on the other hand, has a value of −3 on the general reference factor showing that this individual has a hyperactivity-impulsivity score below average. The scores of this individual on the two specific factors, however, are 0.5 showing that this individual has inattention and oppositional defiant symptoms that are higher than expected given the hyperactivity-impulsivity score. Compared to all other individuals with the same hyperactivity-impulsivity score the inattention and oppositional defiant problems are above average.

**Fig. 2:**
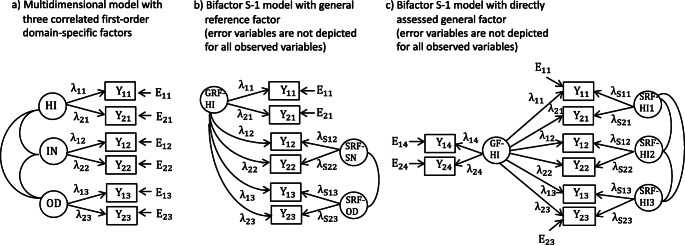
Multidimensional models for analyzing multi-faceted constructs. (**a**) Multidimensional model with correlated first-order facet-specific factors for hyperactivity-impulsivity (HI), inattention (IN), and oppositional defiant disorder (OD). (**b**) Bifactor S-1 model presented by Burns et al. (this issue) with hyperactivity-impulsivity (HI) as general reference factor (GRF-HI) and two specific reference factors for inattention (SRF-IN) and oppositional defiant disorder (SRF-OD). (**c**) Bifactor S-1 model with a directly assessed general hyperactivity-impulsivity factor (GF-HI) and three specific reference factors for hyperactivity-impulsivity assessed in specific situations (SRF-HI1, SRF-HI2, SRF-HI3). Y_ij_: observed variables, E_ij_: error variables, λ_ij_: factor loadings, i: indicator, j: facet

The two examples in Fig. 1a show that the factor scores reveal interesting individual and interindividual differences. It is important that the diagnostic information given by the factor scores are different from the factor scores of the multidimensional CFA model with correlated first-order factors which is a kind of natural starting model for analyzing multi-faceted construct. The basic structure of this model for the three facets of ADHD/ODD is presented in Fig. 2a. In this model, there is a factor for each of the three facets and the factors can be correlated. The factor scores of the two individuals presented in Fig. 1a on the three first-order factors is presented in Fig. 1b. Comparing Figs. 1a und 1b reveals important differences in the diagnostic information between the two models. The factor scores in Fig. 1b indicate whether and to which degree the individual factor scores deviate from the mean of the factor. Because the general reference factor equals the first-order hyperactivity-impulsivity factor, the factor scores on the first factor in Fig. 1a and b are the same. However, the factors scores of Individual A on the first-order factors of inattention and oppositional defiant problems are positive (0.5) showing that the scores of Individual A are above average compared to the *total* sample. Whereas the inattention and oppositional defiant problems of Individual A are below average compared to individuals having the same hyperactivity-impulsivity score they are above average compared to the total sample. Both pieces of information give important insights into the symptom profile of Individual A and complement each other. For Individual B the situation is different. Individual B has scores on all the first-order facets below average and is less affected by these problems than the average of the total sample. However, the scores of Individual B on the specific factors are positive indicating that the severity of inattention and oppositional defiant problems are stronger than average compared to individuals having the same hyperactivity-impulsivity score. Because the information represented by the factor scores give different insights into the severity of psychological symptoms, it is worthwhile to estimate both types of factor scores and use them for psychological assessment.

Because the interpretation of the factor scores (with respect to the deviation from the mean) also depend on the sample it is advisable to apply both the multidimensional model with correlated first-order factors and the bifactor S-1 model to representative samples stemming from interesting norm populations. Because the multidimensional model with correlated first-order factors allows interesting insights and it is more restrictive than the bifactor S-1 model (Geiser et al. 2012) test construction and item selection should be based on the multidimensional model with correlated first-order factors. If the latter model fits the data, then the bifactor S – 1 model will also fit the data. As Geiser et al. have shown, the bifactor S-1 model can be restricted in such a way that it is a reformulation of the multidimensional model with correlated first-order factors and shows the same fit. For individual clinical assessment, it is desirable that the parameters of the bifactor S-1 model do not differ between relevant subgroups to make sure that individual scores can be compared across groups, and a measurement instrument can be broadly applied. Therefore, it is worthwhile to extend the bifactor S-1 model to a multigroup model allowing testing different types of measurement invariance across subgroups (Millsap 2012). Therefore, the study of Burns et al.’s (this issue) can build an important cornerstone for a research program focusing on the suitability of the bifactor S-1 model for clinical assessment.

## Guidelines for Analyzing Multi-Faceted Constructs with Multidimensional Models

**Fig. 3:**
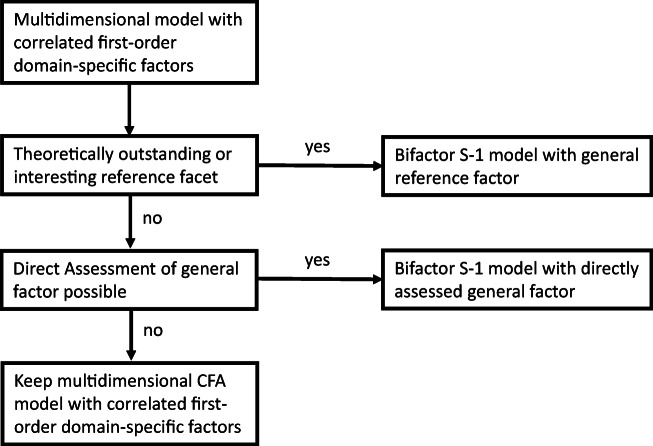
Decision flow chart for selecting an appropriate model for analyzing multi-faceted constructs

A strong merit of Burns et al.’s (this issue) application of the bifactor S-1 model is that they select the reference facet in a convincing way based on theoretical assumptions and empirical studies on the development of ADHD/ODD symptoms. Not in all areas of clinical research such an outstanding facet might exist. How can this problem be solved? How can multi-faceted constructs be generally analyzed? In the following some general guidelines and decision rules will be presented (see Fig. 3):

1. A starting point for analyzing multi-faceted construct is the multidimensional model with correlated first-order factors. This model represents the idea that there are multiple distinct (non-overlapping) facets of a construct. This model is an appropriate model for test construction and items selection. The number and meaning of the different facets depend on the area of interest and should be guided by theoretical assumptions about the construct under consideration.
2. If there is a theoretically outstanding facet – like in the analysis of Burns et al. (this issue) – this facet can be taken as reference facet and a bifactor S-1 can be defined with the items of the reference facet defining the general reference factor (Fig. 2b).
3. If there is no theoretically outstanding facet the researcher can decide whether there is a reference facet of special interest. Consider, for example, a researcher is assessing hyperactivity-impulsivity in three different types of situations: (1) at school, (2) at home, and (3) during sports and exercise. From a theoretical point of view there might not be a class of situations that is superordinate to other classes of situations. However, a researcher might be interested in comparing ADHD/ODD symptoms at home to ADHD/ODD symptoms in situations outside the home. In this case, the items assessing ADHD/ODD symptoms at home would indicate the general reference factor and the scores on the specific factors would indicate to which degree an individual shows more intense or less intense symptoms at schools and during sports and exercise compared to what one expects based on the ADHD/ODD symptoms at home.
4. If there is no outstanding facet but strong assumptions about a reasonable general factor, the general factor can be directly assessed (see Fig. 2c). The model in Fig. 2c is also a bifactor S-1 model but with the directly assessed general factor as reference factor. If, for example hyperactivity-impulsivity is assessed with respect to the three classes of situations (at school, at home, during sports and exercise) one could add items assessing ADHD/ODD symptoms in general. One could, for example, present the same items with four different instructions assessing the symptoms in general (like the scales used in the study of Burns et al. this issue) and assessing the symptoms in the three different situations. In such an application, the general factor also has a clear meaning (symptoms in general). Moreover, the specific factors indicate to which degree the symptoms in the three situations differ from what can be expected given the general assessment.
5. If there is no theoretically outstanding facet and no facet of special interest and if the general factor cannot be directly assessed one can retain the multidimensional model with correlated first-order factors as the most appropriate model and can present profiles of individual scores like the profiles presented in Fig. 1b. For example, in the study of Burns et al. (this issue) academic and social impairment is assessed by three factors (social impairment, academic impairment, peer rejection). In this case, the direct assessment of a general factor might not be possible because it might not be clear what such a factor would mean and how it can be defined by appropriate indicators. If a researcher does not want one of the three impairment factors as an appropriate comparison standard, the multidimensional model with correlated first-order factors might be appropriate.

Even if a variant of the bifactor S-1 will be specified from a theoretical point of view it is advisable to also report the results of the multidimensional CFA model with correlated first-order factors. From the perspectives of individual clinical assessment, the factor scores of both types of models (multidimensional model with correlated first-order factor, bifactor S-1 model) are interesting and should be reported (with confidence intervals). The flow chart in Fig. 3 does not mean that one has to strictly follow this strategy. For example, it could be interesting to apply all three models presented in Fig. 2 in an empirical study combining the advantages of these models.

## Summary and Discussion

Burns et al. (this issue) show that the application of the symmetrical bifactor model to ADHD/ODD symptoms reveals problematic and inconsistent results that indicate that the symmetrical bifactor is not an appropriate model for analyzing this type of symptoms and can not be used as a psychometric basis for individual clinical assessment. In contrast, applications of the bifactor S-1 model do not show this problems and reveal consistent results. Moreover, Burns et al. show that there is a theoretically outstanding reference facet that gives the factors of the bifactor S-1 a clear and interesting meaning. This makes it possible to use the bifactor S-1 model for individual clinical assessment. The factor scores have a clear meaning. The factor scores on the general reference factor indicate the severity of individual clinical problems compared to the total sample. The factor scores on the specific factors compare the individual clinical symptoms to the distribution of the individuals having the same score on the general reference factor. This is important information that complement the information given by the factor scores on the first-order factors of a multidimensional CFA model. Both diagnostic information are interesting and it is therefore advisable to report both types of factor scores.

A general decision flow chart for selecting an appropriate model for analyzing multi-faceted constructs was presented. Starting with a multidimensional model with correlated first-order factors a researcher can decide whether a bifactor S-1 model with a general reference factor or with a directly assessed general factor would be an appropriate model. Both models (with a general reference factor and with a directly assessed general factor) reveal important information in addition to the basic multidimensional model with correlated first-order factors, which should always be reported.

Given the superiority of the bifactor S-1 model over the bifactor model for the analysis of multi-faceted clinical symptoms (Burns et al. this issue; Heinrich et al. 2018), it is worthwhile to start a research program on the feasibility of the bifactor S-1 model for individual clinical assessment. Currently there are too few applications of this model in clinical psychology, and more empirical studies are needed.
